# Optimal Positive End Expiratory Pressure Levels in Ventilated Patients Without Acute Respiratory Distress Syndrome: A Bayesian Network Meta-Analysis and Systematic Review of Randomized Controlled Trials

**DOI:** 10.3389/fmed.2021.730018

**Published:** 2021-09-01

**Authors:** Jing Zhou, Zhimin Lin, Xiumei Deng, Baiyun Liu, Yu Zhang, Yongxin Zheng, Haichong Zheng, Yingzhi Wang, Yan Lai, Weixiang Huang, Xiaoqing Liu, Weiqun He, Yuanda Xu, Yimin Li, Yongbo Huang, Ling Sang

**Affiliations:** ^1^State Key Laboratory of Respiratory Disease, National Clinical Research Center for Respiratory Disease, Guangzhou Institute of Respiratory Health, The First Affiliated Hospital of Guangzhou Medical University, Guangzhou, China; ^2^Guangzhou Laboratory, Guangdong, China

**Keywords:** Acute respiratory distress syndrome, Mechanical ventilation, Positive end expiratory pressure, Pneumothorax, Mortality

## Abstract

**Background:** To find the optimal positive end expiratory pressure (PEEP) in mechanical ventilated patients without Acute Respiratory Distress Syndrome (ARDS), we conducted a Bayesian network meta-analysis and systematic review of randomized controlled trials (RCTs) comparing different level of PEEP based on a novel classification of PEEP level: ZEEP group (PEEP = 0 cm H2O); lower PEEP group (PEEP = 1–6 cm H2O); intermediate PEEP group (PEEP = 7–10 cm H2O); higher PEEP group (PEEP > 10 cm H2O).

**Result:** Twenty eight eligible studies with 2,712 patients were included. There were no significant differences in the duration of mechanical ventilation between higher and intermediate PEEP (MD: 0.020, 95% CI: −0.14, 0.28), higher and lower PEEP (MD: −0.010, 95% CI: −0.23, 0.22), higher PEEP and ZEEP (MD: 0.010, 95% CI: −0.40, 0.22), intermediate and lower PEEP (MD: −0.040, 95% CI: −0.18, 0.040), intermediate PEEP and ZEEP (MD: −0.010, 95% CI: −0.42, 0.10), lower PEEP and ZEEP (MD: 0.020, 95% CI: −0.32, 0.13), respectively. Higher PEEP was associated with significantly higher PaO2/FiO2 ratio(PFR) when compared to ZEEP (MD: 73.24, 95% CI: 11.03, 130.7), and higher incidence of pneumothorax when compared to intermediate PEEP, lower PEEP and ZEEP (OR: 2.91e + 12, 95% CI: 40.3, 1.76e + 39; OR: 1.85e + 12, 95% CI: 29.2, 1.18e + 39; and OR: 1.44e + 12, 95% CI: 16.9, 8.70e + 38, respectively). There was no association between PEEP levels and other secondary outcomes.

**Conclusion:** We identified higher PEEP was associated with significantly higher PFR and higher incidence of pneumothorax. Nonetheless, in terms of other outcomes, no significant differences were detected among four levels of PEEP.

**Systematic Review Registration:** The study had registered on an international prospective register of systematic reviews, PROSPERO, on 09 April 2021, identifier: [CRD42021241745].

## Introduction

Although invasive mechanical ventilation is a lifesaving strategy for critically ill patients, previous studies have considered it a potentially harmful intervention (1, 2). Positive end expiratory pressure (PEEP) has shown efficacy in maintaining alveoli opening, improvement of gas exchange and reduction of injurious shear forces in acute respiratory distress syndrome (ARDS) patients since 1960 s (3). To date, however, the optimal PEEP levels remain unclear, owing to occurrence of potential negative effects that cause overdistention of the lungs, exacerbate lung stress as well as strain and impair hemodynamics by reducing venous return and increasing pulmonary vascular resistance. Therefore, PEEP's net benefits or harm are depended on the balance between alveolar recruitment and overdistension, and should be particularly beneficial in disease states with substantial alveolar collapse (4). Nevertheless, this trade-off is often difficult to achieve clinically.

Similarly, the optimal PEEP level for mechanical ventilated patients without ARDS remains unclear. Several studies have demonstrated that higher PEEP levels could improve oxygenation, reduce occurrence of ventilator-associated pneumonia (VAP), prevent ARDS in this population (5). In fact, application of PEEP has increased in clinical practice (6). However, PEEP level in a relatively healthy lung is expected to be lower because of less lung collapse which requires less pressure to open the collapsed lung. In addition, previous research evidences from animal studies have shown that ventilation with higher PEEP levels might worsen existing lung injuries or cause development of new ones (7–9). A recent RELAx trial demonstrated that a higher PEEP strategy generated clinically superior outcomes than lower levels with regards to the number of ventilator-free days (VFD) at day 28 in ventilated patients without ARDS, although there was a possibility of elevated hypoxemia in the lower PEEP group (10).

A previous systematic review and meta-analysis compared efficacy of different PEEP levels in patients without ARDS (11). However, the findings therein should be interpreted with caution, owing to a moderate to high heterogeneity, a low to very low quality of evidences (QoE), and the fact that the included studies could not allow the authors to comprehensively address the effects of moderate PEEP levels. In the present study, we conducted a Bayesian network meta-analysis and systematic review of RCTs to compare efficacy of different PEEP levels in ventilated patients without ARDS, and identify the optimal level for this population. Specifically, we divided the patients into four groups according to their PEEP levels. We chose a novel classification, based on patients' PEEP levels, which is closer to clinical practice, and can allow for reduction of heterogeneity in the analysis as well as precise evaluation of the effects of different PEEP levels.

## Materials and Methods

This meta-analysis was performed in accordance with the guidelines of the Preferred Reporting Items for Systematic Reviews and Meta-analyses extension statement for reporting network meta-analyses (PRISMA- NMA) (12). The study was also prospectively registered on PROSPERO database (Registration number: CRD42021241745).

### Data Sources and Study Search

We searched PubMed, Web of Science, Embase, Cochrane Library, Embase up to January 2021. Reference lists of relevant articles were also reviewed. The inclusion criteria were as follows: (i) studies were RCTs; (ii) the study population comprised ventilated patients without ARDS; (iii) intervention included higher *vs*. lower PEEP; and (iv) studies were published in English. The exclusion criteria were as follows: (i) studies that analyzed pediatric patients; (ii) patients were not in ICU; (iii) data were unavailable; and (iv) duplicate publications.

### Study Selection and Data Extraction

Meta-analysis was performed by two researchers (JZ and ZML), who independently screened the citations and abstracts in duplicate and extracted the data. All references that were judged potentially relevant were evaluated for full-text eligibility. Discrepancies were resolved by consensus with a third author (YBH). In cases where relevant data or information was missing, we attempted to contact the authors of the studies.

### Outcome Measures

Primary outcome was the duration of mechanical ventilation, whereas secondary outcomes included PaO_2_/FiO_2_ ratio (PFR), length of stay (LOS) in ICU, LOS in hospital, hospital mortality, 28-day mortality, ICU mortality, occurrence of ARDS, pneumothorax, atelectasis and hypoxemia.

### Assessment of Risk of Bias

Two authors (JZ and ZML) independently assessed the risk of bias (RoB) in individual studies, using the revised Cochrane risk-of-bias tool for randomized trials (13), and classified them as either low or high. Any disagreements between them were resolved by discussion and consensus with a third author (YBH). Low-biased studies were defined as those with no <4 low-risk items, based on the Cochrane risk-of-bias tool.

### Statistical Analysis

A random effects network meta-analysis was performed using a Bayesian framework. We also calculated mean differences for continuous outcomes and odds ratios (ORs) for dichotomous outcomes, then converted medians and interquartile ranges to means and standard deviations as previously described (14). Network meta-analysis was performed using the “gemtc” package (version 0.8–2) implemented in R version 3.4.4 (https://www.r-project.org/). This package is based on an approach that follows the graph-theoretical methodology. We ranked the treatments using the P-score to reveal the degree of certainty that a specific treatment was better than the others. Based on this, P-scores close to 1 and 0 denoted the best and worst treatments, respectively. Moreover, studies followed by a value of *I*^2^ ≥ 50% were considered to have substantial heterogeneity. To limit the possibility of type I error, we performed a Trial sequential analysis (TSA) using TSA version 0.9.5.10.

## Results

### Eligible Studies

**Figure 1:**
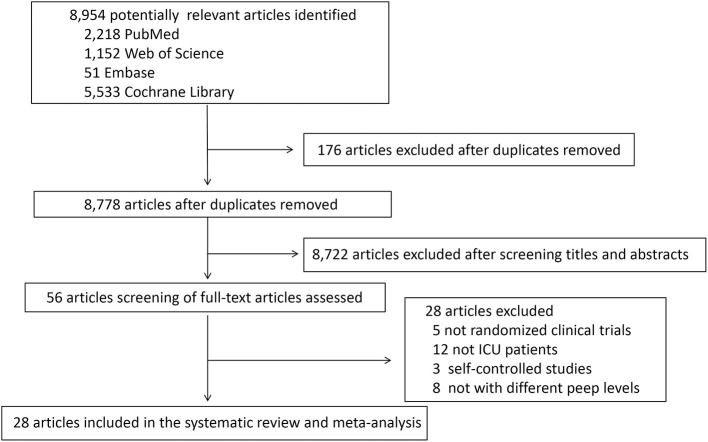
PRISMA Flowchart.

A total of 8,954 articles were retrieved from the aforementioned databases, of which 56 were considered potentially eligible after reviewing their full texts. Finally, 28 studies (5, 10, 15–40), comprising 2,712 patients, met all our inclusion criteria and were included in the meta-analysis (Figure 1).

**Figure 2:**
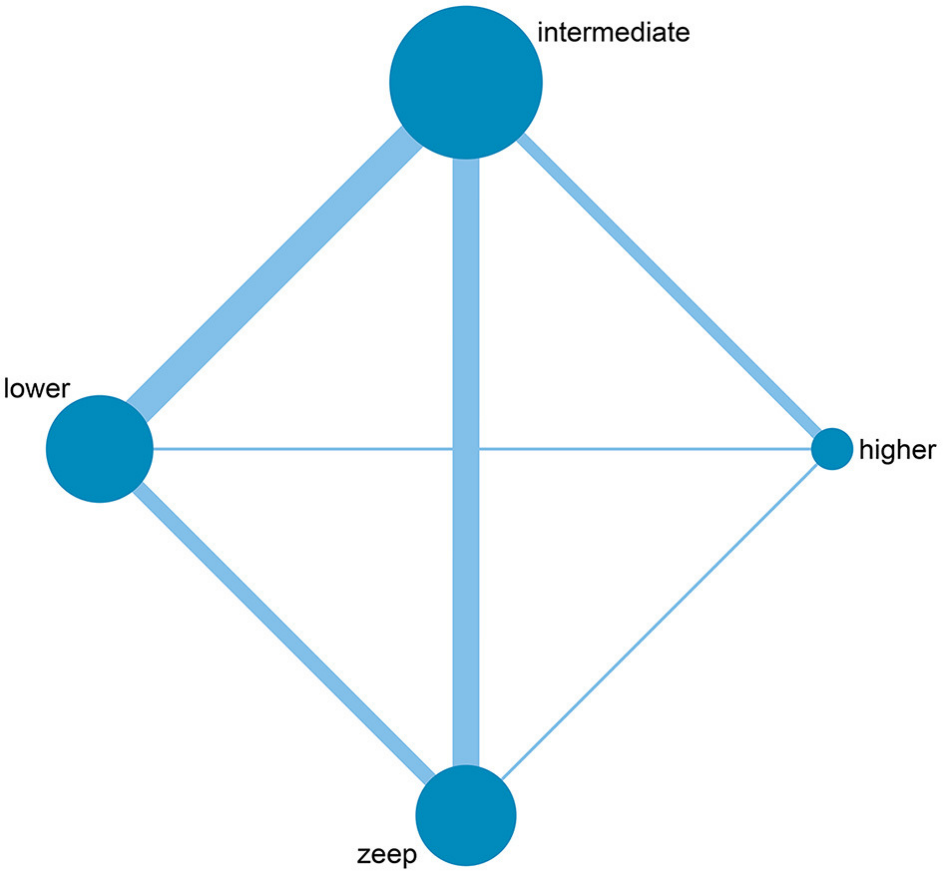
Network geometry of included studies. The size of the nodes is proportional to the number of patients randomized to receive the intervention. The width of the lines is proportional to the number of trials comparing the connected intervention.

**Figure 3:**
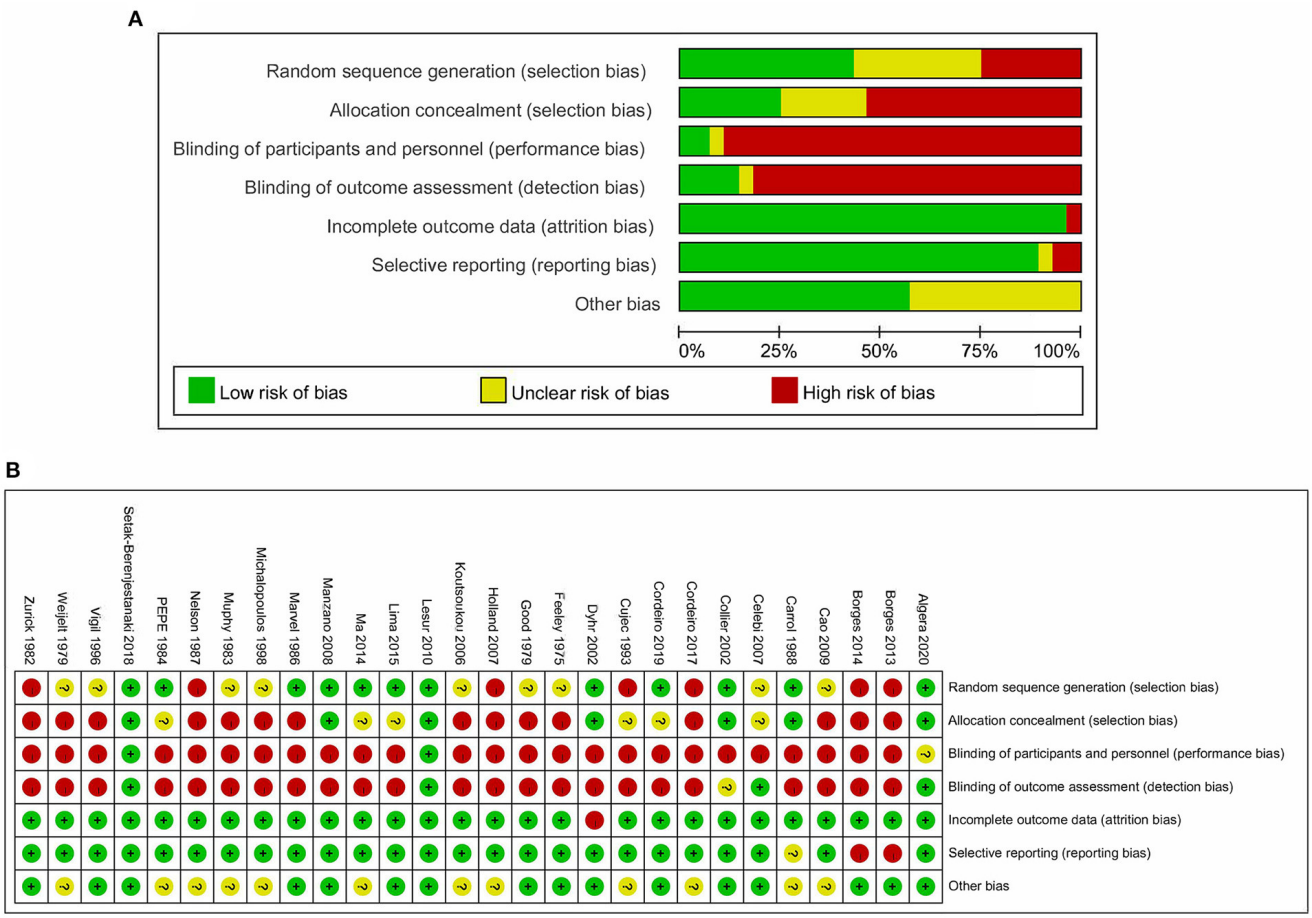
Risk of bias of included studies. **(A)** Risk of bias graph based on the Cochrane Risk of Bias Tool. **(B)** Risk of bias summary based on the Cochrane Risk of Bias Tool.

Among the 28 eligible trials, 1 compared higher PEEP levels with ZEEP, 7 compared intermediate PEEP with ZEEP, 4 compared lower PEEP with ZEEP, 1 compared higher with lower PEEP, 4 compared higher with intermediate PEEP, 8 compared intermediate with lower PEEP, while 3 compared intermediate with lower PEEP and ZEEP. Sample sizes in these trials ranged from 15 to 969 patients. The network geometry is shown in Figure 2. With regards to regions, the eligible RCTs were conducted across different countries in the world, with 16 of them focusing on post-cardiac patients. Meanwhile, the year of publication widely varied across the studies, with 12 of them published before 2000 (Table 1). RoB was high in 18 (15–17, 20, 23, 24, 27, 28, 30–32, 34–38, 40, 41) and low in 10 (5, 10, 18, 19, 21, 22, 25, 26, 29, 33) trials. The high RoB was attributed to blinding of participants, personnel and outcome assessors (Figure 3).

**Table 1:** Characteristics of included studies.

**Study;** **country**	**Type of patients;** **Mean age (years)**	**N**	**Interventions**	**Sample** **Size**	**TV** **(ml/kg)**	**RM**	**Main findings**
**Post-Cardiac Surgery Patients**							
Borges et al. (15); Brazil	Post-CABG; 60	136	M vs. L	92/44	6~8	NO	Better pulmonary compliance values, oxygenation indexes, and lower frequency of hypoxemia were found in higher PEEP group
Lago Borges et al. (16); Brazil	Post-CABG; 60	136	M vs. L	92/44	6~8	NO	Patients in higher PEEP group had shorter duration of mechanical ventilation.
Carroll et al. (17); America	Postoperative; 63	50	H vs. L	22/28	12	YES	Higher incidence of barotrauma and hypotension and death and higher duration of ventilation with higher PEEP.
Celebi et al. (18); Turkey	Post-CABG; 56	40	M vs. L	20/20	7	YES	Higher P/F ratio in the first 4h and less atelectasis in higher PEEP group.
Collier et al. (19); America	Post-cardiac surgery; 66	84	M vs. L	40/44	10	NO	Higher PEEP does not decrease chest-tube output or transfusion requirements but it may increase the fluid requirements.
Cordeiro et al. (20); Brazil	Post-CABG; 61	30	H vs. M	20/10	6~8	NO	Non-invasive ventilation with PEEP 15cm H2O represented an improvement in oxygenation levels.
Cordeiro et al. (21); Brazil	Post-cardiac surgery; 64	60	H vs. M	41/19	6	NO	Significant improvement in the oxygenation rate with higher peep.
Dyhr et al. (22); Denmark	Post-CABG; 60	15	H vs. Z	7/8	6	YES	Improvement in P/F ratio and end-expiratory lung volume in PEEP group.
Good et al. (23); America	Post-cardiac surgery; 55	24	M vs. Z	10/14	10~12	NO	Routine PEEP did not prevent atelectasis or improve pulmonary oxygen transport.
Holland et al. (24); Germany	Post-cardiac surgery; 66	28	M vs. L	14/14	6~8	NO	A PEEP of 10 mbar over 2 h did not compromise liver function and gastric mucosal perfusion
Lima et al. (25); Brazil	Post-CABG; 62	78	M vs. L	46/32	6~8	NO	No difference in gas exchange in the first 6 h after extubation between groups.
Marvel et al. (26); America	Post-CABG; 59	44	M vs. L vs. Z	12/15/17	NA	NO	No difference in the incidence of atelectasis or duration of hospitalization among groups.
Michalopoulos et al. (27); Greece	Post-CABG; 61	67	M vs. L vs. Z	21/24/22	NA	No	No differences in PaO_2_/FiO_2_, SvO_2_, PvO_2_ and in cardiac index among the three groups
Murphy et al. (28); America	Post-cardiac surgery; NA	139	M vs. Z	NA	NA	NO	PEEP reduced mediastinal bleeding after cardiac operations
Setak-Berenjestanaki et al. (29); Iran	Post-cardiac surgery; 56	180	M vs. L	120/60	NA	NO	Higher peep resulted in lower incidence of atelectasis and shorter duration of intubation
Zurick et al. (30); America	Post-cardiac surgery; 57	83	M vs. Z	41/42	NA	NO	PEEP did not reduce the amount of blood loss, the need for reexploration for bleeding, or the blood requirements
**Non-Post-Cardiac Surgery Patients**							
Cujec et al. (31); Canada	ARF: 59	46	M vs. Z	NA	NA	NO	Higher PEEP reduced alveolar–arterial oxygen difference and shunt fraction
Koutsoukou et al. (32); Greece	Severe brain damage; 41	21	M vs. Z	11/10	8~10	NO	Five days of mechanical ventilation on ZEEP resulted in higher static elastance and minimal resistance
Lesur et al. (33); Canada	ARF; 64	63	L vs. Z	30/33	6~9	NO	No difference in the occurrence of hypotension and duration of ventilation and mortality
Ma et al. (31); China	NPE; 64	120	H vs. M	60/60	6~8	NO	Higher PEEP resulted in lower 28-day morality rate and higher P/F ratio
Nelson et al. (35); America	At risk of ARF; 54	38	H vs. M	20/18	NA	NO	No difference in entry PaO2, intubated/ICU/hospitalization days, incidence of barotrauma, ICU/overall mortality between groups.
Pepe et al. (36); America	At risk of ARDS; 44	92	M vs. Z	44/48	12	NO	No difference in the incidence of the ARDS or other associated complications between groups.
Vigil et al. (37); America	Trauma; 34	44	L vs. Z	23/21	12~15	NO	Significantly less hospitalization days in zeep group whereas higher P/F ratio in the peep group.
Weijelt et al. (38); America	At risk of ARDS; 45	79	L vs. Z	45/34	15	NO	Peep altered the degree of deterioration and incidence of ARDS rather than preventing its occurrence
**Miscellaneous Patients**							
Algera et al. (10); Netherlands	Receiving IMV; 66	969	M vs. L	493/476	6~8	NO	With regard to the number of ventilator-free days at day 28, no difference was found between the two groups
Cao et al. (39); China	Hypovolemic patients; 44	30	M vs. L vs. Z	10/10/10	6~8	NO	Higher levels of PEEP increased CVP and CIVP
Manzano et al. (5); Spain	Without hypoxemia; 45	127	M vs. Z	64/63	8~9	NO	Application of prophylactic PEEP reduced the number of hypoxemia episodes and the incidence of ventilator-associated pneumonia
Feeley et al. (40); America	ARF; 61	25	L vs. Z	12/13	10	NO	PEEP may be useful in weaning patients who have a low vital capacity and inspiratory force

*N means total number of participants in each study; Sample Size means number of participants in each group in study; NA, not available; TV, tidal volume; RM, recruitment maneuvers; PEEP, positive end-expiratory pressure (in cmH2O); P/F ratio: PaO2/FiO2; IMV, invasive mechanical ventilation; CABG, coronary artery bypass grafting; ARF, acute respiratory failure; ARDS, acute respiratory distress syndrome; NPE, neurological pulmonary edema.*

*H, higher peep (peep level >10 cmH_2_0); M, intermediate peep (5 < peep level ≤ 10 cmH_2_0); L, lower peep (0 < peep level ≤ 5 cmH_2_0); Z, zeep means peep level of zero*.

### Primary Outcomes

**Figure 4:**
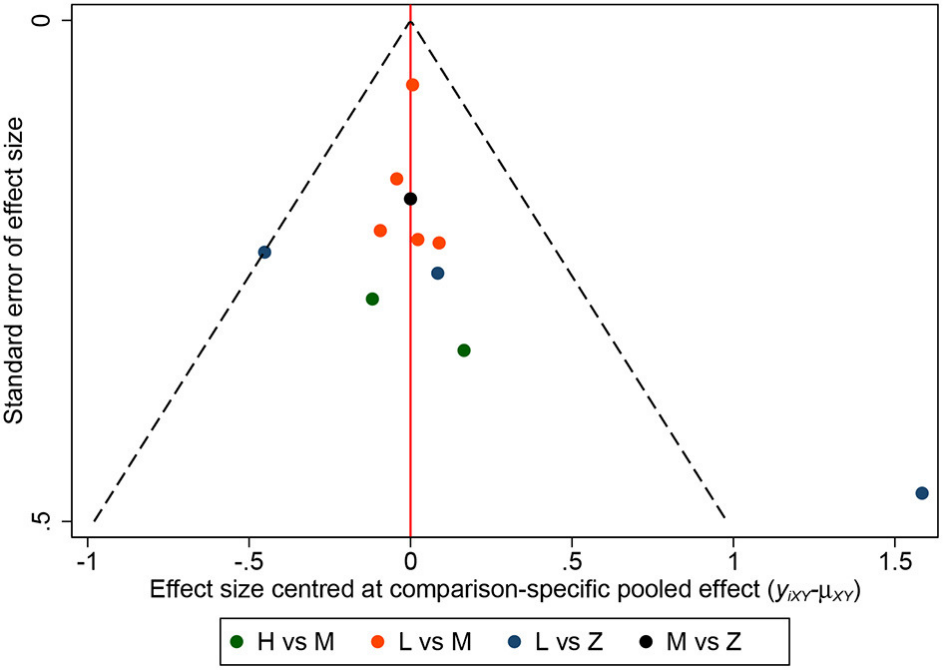
Funnel plot for duration of mechanical ventilation. Funnel plot of association between estimated effect size for each study in terms of duration of mechanical ventilation.

**Figure 5:**
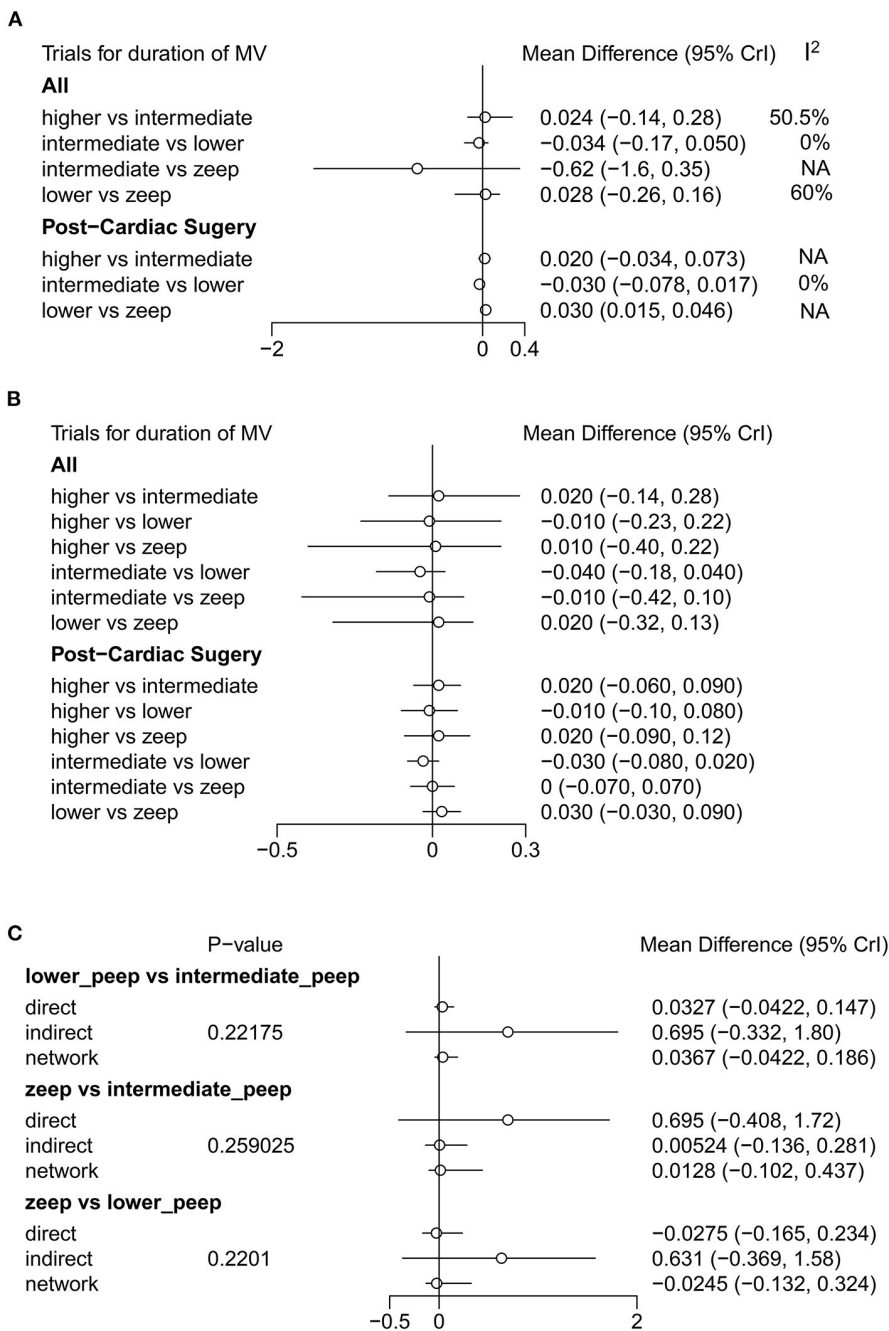
Forest plot of duration of mechanical ventilation. **(A)** Results of direct comparison and heterogeneity test. **(B)** Results of Network Meta-Analysis. **(C)** Node-splitting analysis to assess inconsistency in network meta-analysis. In addition to general population (No statistic difference in inconsistency between direct result and indirect result when *P*-value > 0.05), **(A,B)** also show the results of analysis among post-cardiac surgery patients.

**Figure 6:**
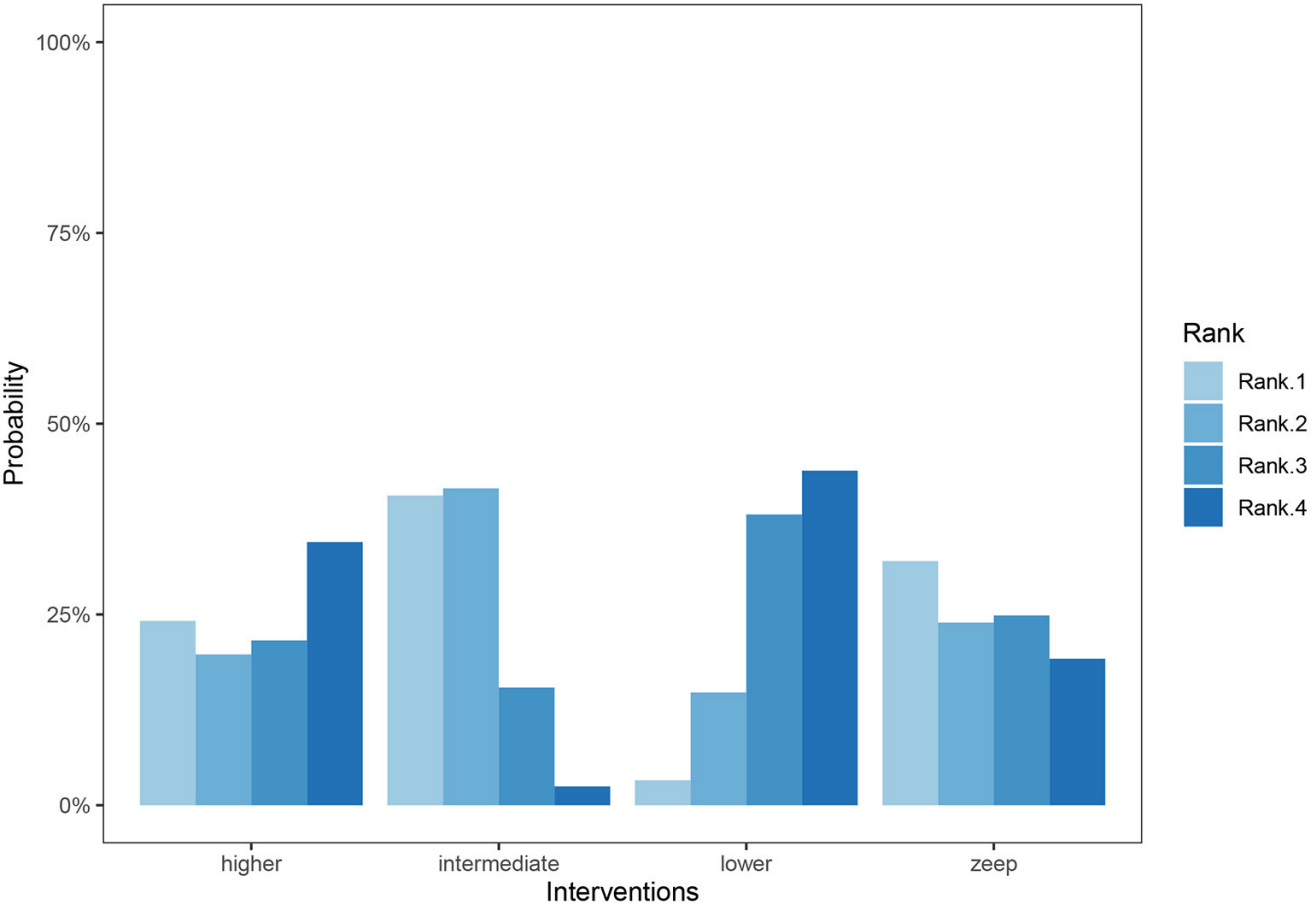
Cumulative ranking bar graph for duration of mechanical ventilation. Ranks represent priority. For each intervention, cumulative ranking bar graph shows the probabilities when they are at Rank1/2/3/4 respectively. To sum up, the probabilities of every 4 columns in each intervention are 100%.

**Figure 7:**
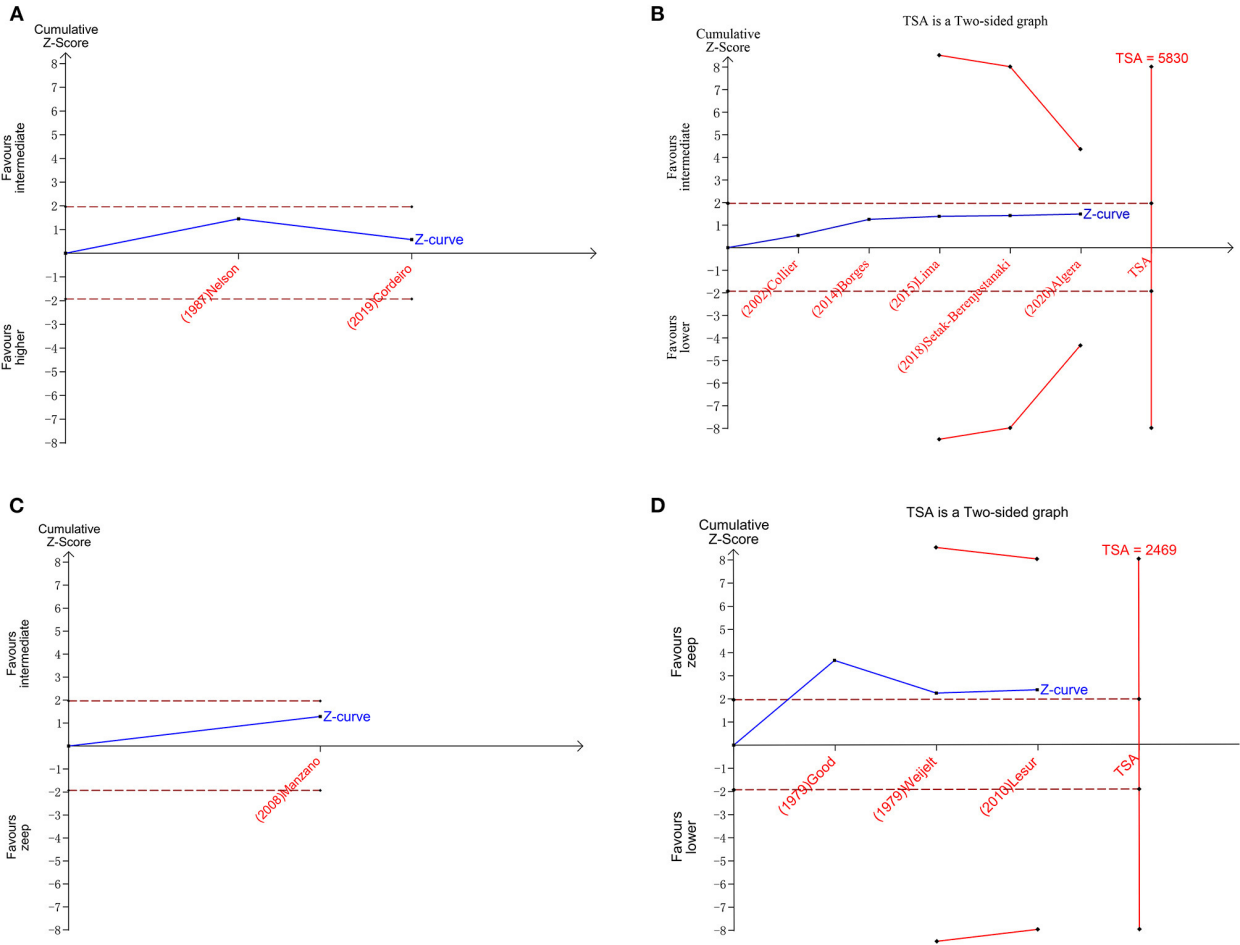
Plots of trial sequential analysis (TSA) for duration of mechanical ventilation. **(A)** TSA for higher peep *vs*. intermediate peep; **(B)** TSA for intermediate peep vs. lower peep; **(C)** TSA for intermediate peep vs. Zeep; **(D)** TSA for lower peep vs. Zeep. TSA boundary is ignored due to too little information use in **A,C**.

A total of 11 eligible articles (5, 10, 16, 19, 21, 23, 25, 29, 33, 35, 38), with 1,848 participants, reported duration of mechanical ventilation. Among them, 6 studies (16, 19, 21, 23, 25, 29), with 572 patients (16, 19, 21, 23, 25, 29). A summary of the RoBs is shown in Figure 3 while the resulting funnel plot is illustrated in Figure 4. A direct comparison revealed no significant differences in the duration of mechanical ventilation, between higher and intermediate PEEP levels (MD: 0.024, 95% CI: −0.14, 0.28), intermediate and lower PEEP (MD: −0.034, 95% CI: −0.17, 0.050), intermediate PEEP and ZEEP (MD: −0.62, 95% CI: −1.6, 0.35), as well as lower PEEP and ZEEP (MD: −0.028, 95% CI: −0.26, 0.16). Similarly, a direct comparison among a subpopulation of post-cardiac surgery patients revealed no significant differences in the duration of mechanical ventilation among different PEEP strategies (higher vs. intermediate: MD: 0.02, 95% CI: −0.034, 0.073; intermediate vs. lower: MD: −0.03, 95% CI: −0.078, 0.017; and lower PEEP *vs*. ZEEP: MD: 0.03, 95% CI: 0.015, 0.046) (Figure 5A). Results from Network Meta-Analysis, which combined direct and indirect comparison approaches, revealed no significant differences in the duration of mechanical ventilation between higher and intermediate PEEP (MD: 0.020, 95% CI: −0.14, 0.28), higher and lower PEEP (MD: −0.010, 95% CI: −0.23, 0.22), higher PEEP and ZEEP (MD: 0.010, 95% CI: −0.40, 0.22), intermediate and lower PEEP (MD: −0.040, 95% CI: −0.18, 0.040), intermediate PEEP and ZEEP (MD: −0.010, 95% CI: −0.42, 0.10), as well as lower PEEP and ZEEP (MD: 0.020, 95% CI: −0.32, 0.13) groups. Pooled estimates from the network meta-analysis were shown in Table 2. Network Meta-Analysis of the subpopulation of post-cardiac surgery patients also revealed no significant differences in their duration of mechanical ventilation among different PEEP strategies (higher vs. intermediate PEEP: MD: 0.02, 95% CI: −0.060, 0.090; higher vs. lower PEEP: MD: −0.010, 95% CI: −0.10, 0.080; higher PEEP vs. ZEEP: MD: 0.02, 95% CI: −0.090, 0.12; intermediate *vs*. lower PEEP: MD: −0.03, 95% CI: −0.080, 0.020; intermediate PEEP vs. ZEEP: MD: 0, 95% CI: −0.070, 0.070; lower PEEP vs. ZEEP: MD: 0.03, 95% CI: −0.030, 0.090) (Figure 5B). We also performed node-splitting analysis to assess inconsistency in network meta-analysis, and found no significant differences between intermediate *vs*. lower PEEP (*p* = 0.22), intermediate PEEP vs. ZEEP (*p* = 0.26), and lower PEEP vs. ZEEP (*p* = 0.22), indicating that the results from both direct and indirect comparisons across the three groups were highly consistent (Figure 5C). However, results from ranking analysis showed that intermediate PEEP levels could shorten the duration of mechanical ventilation, followed by ZEEP, higher PEEP and lower PEEP (Figure 6). Furthermore, TSA showed that conventional and O'Brien-Fleming significance boundaries were not crossed by the cumulative Z-curve, indicating that the evidence was not sufficient and conclusive. Therefore, further trials are needed to validate these findings. A graphical representation of this analysis is shown in Figure 7.

**Table 2:** Pooled estimates of the network meta-analysis for “duration of MV.”

**Relative effects**	**Higher**	**Intermediate**	**Lower**	**Zeep**
Higher	–	−0.02 (−0.28, 0.14)	0.01 (−0.22, 0.23)	−0.01 (−0.22, 0.40)
Intermediate	0.02 (−0.14, 0.28)	–	0.04 (−0.04, 0.18)	0.01 (−0.10, 0.43)
Lower	−0.01 (−0.23, 0.22)	−0.04 (−0.18, 0.04)	–	−0.02 (−0.13, 0.32)
Zeep	0.01 (−0.40, 0.22)	−0.01 (−0.43, 0.10)	0.02 (−0.32, 0.13)	–

*Results are MDs in the column-defining treatment compared with MDs in the row-defining treatment. Given that “duration of MV” is a negative outcome, MD <0 favored the column-defining treatment. MD, mean difference; MV, mechanical ventilation*.

### Secondary Outcomes

**Figure 8:**
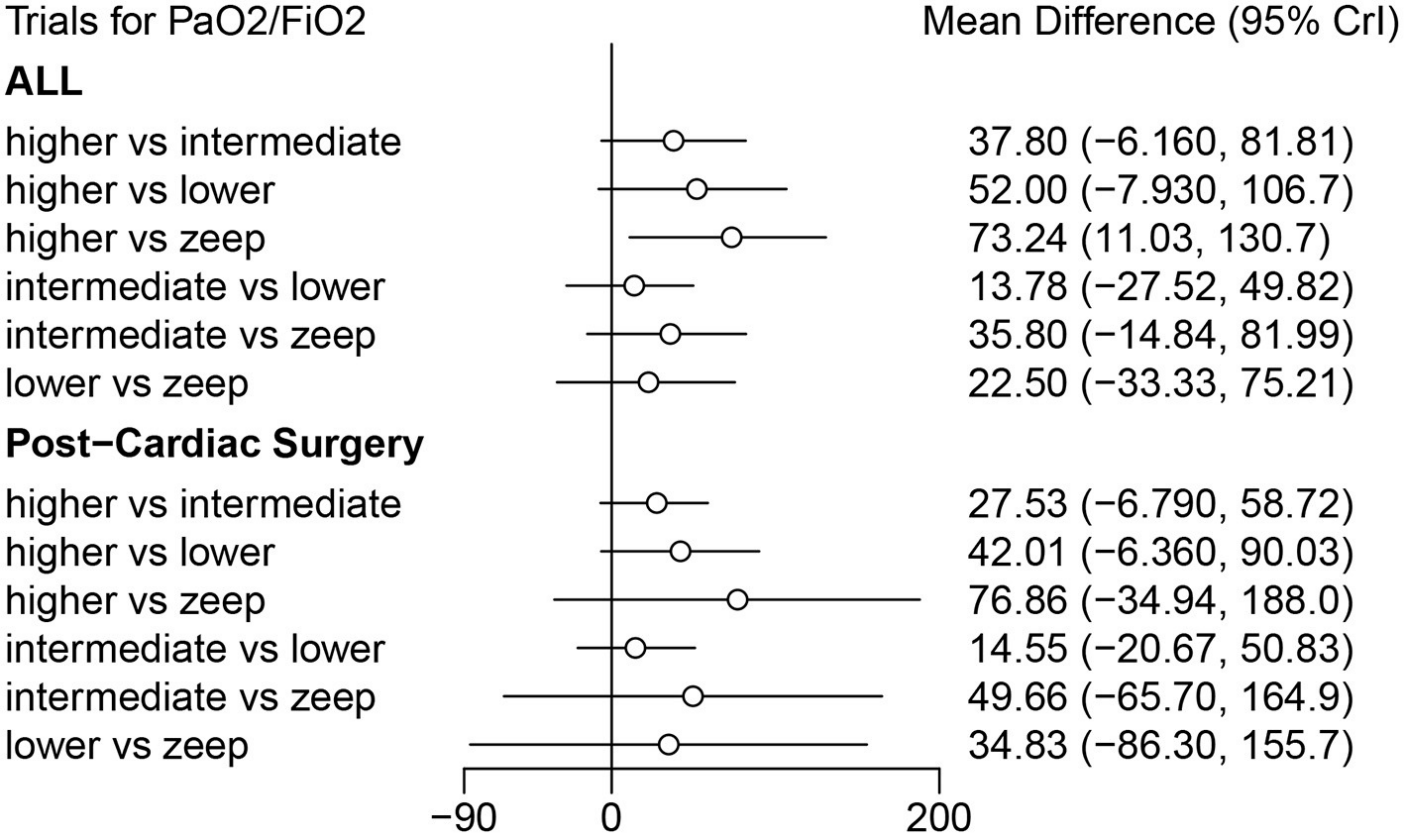
Forest plot of Network Meta-Analysis for PaO_2_/FiO_2_.

Eleven eligible studies, with 1,648 patients, reported on PFR (5, 10, 15, 20–22, 24, 25, 32–34), with 6 of them (comprising 347 patients) focusing on post-cardiac surgery patients (5, 10, 21, 22, 24, 33). Results of RoB are shown in Figure 3 and Supplementary Figure 1A. Direct comparison revealed no significant differences in PFR among PEEP levels, in both general or post cardiac surgical patients (Supplementary Figure 1B). However, results from Network Meta-Analysis demonstrated that higher PEEP was associated with significantly higher PFR compared to ZEEP in the general population (MD: 73.24, 95% CI: 11.03, 130.7). Meawhile, there were no sigificant differences based on the other comparisons (Figure 8). Moreover, node-splitting analysis, based on both direct and indirect comparisons in these groups, revealed consistent results (all *p* > 0.05) (Supplementary Figure 1C). Ranking analysis showed that higher PEEP was associated with the best PFR, followed by intermediate and lower PEEP, and lastly ZEEP (Supplementary Figure 1D).

A total of 7 studies (5, 10, 18, 19, 26, 29, 35), comprising 1,482 patients, reported LOS of hospital, with 4 (18, 19, 26, 29) of them (that analyzed 348 patients) focusing on post-cardiac surgery patients. Direct comparisons and Network Meta-Analysis revealed no significant differences among all PEEP levels in either the general or post cardiac surgical patients (Supplementary Figure 2). Additionally, 6 studies (5, 10, 18, 29, 35, 38) (with 1,433 patients) reported LOS of ICU, with direct comparison showing that lower PEEP levels were associated with shorter LOS of ICU relative to ZEEP (MD: −6.00, 95% CI: −9.80, −2.20) (Supplementary Figure 3A). However, Network Meta-Analysis revealed no significant differences among all PEEP levels (Supplementary Figure 3B). Hospital mortality was reported in 9 eligible studies (5, 10, 17, 19, 27, 33, 35, 36, 38), comprising 1,561 patients, 28-days mortality was reported in 3 eligible studies (10, 33, 34), that analyzed 1,152 patients, while ICU mortality was reported in 3 eligible studies (10, 17, 35) (with 1,056 patients). Notably, only direct comparison showed that higher PEEP levels were associated with increased ICU mortality when compared to lower PEEP (OR: 10.1, 95% CI: 1.21, 91.9) (Supplementary Figure 4A). Results from Network Meta-Analysis revealed no significant differences among the PEEP levels with regards to hospital, 28 days and ICU mortality (Supplementary Figure 4B).

**Figure 9:**
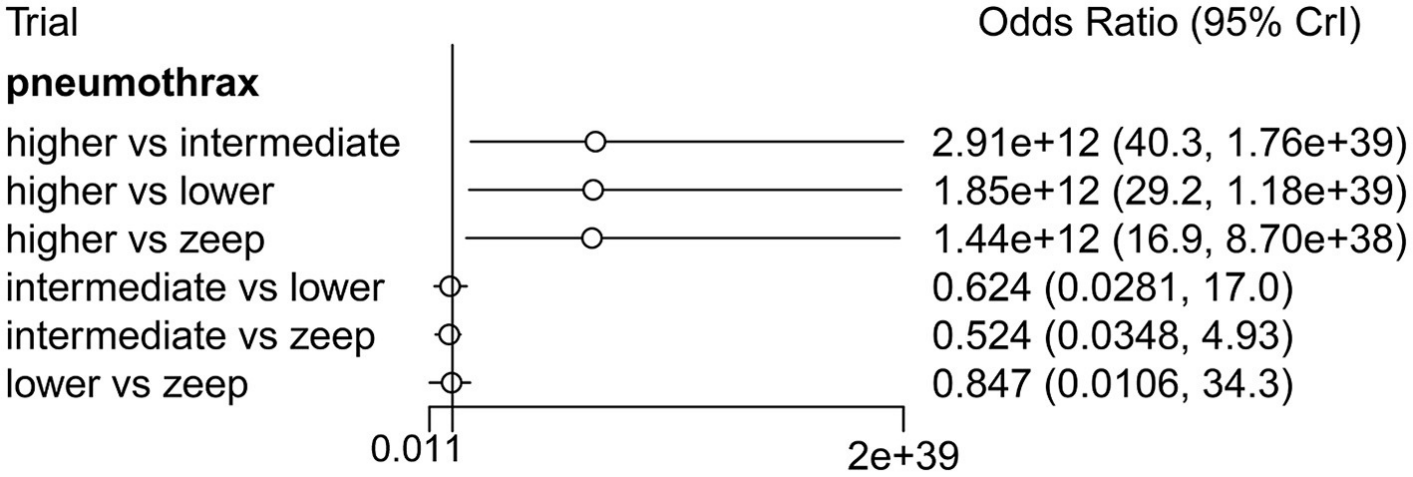
Forest plot of Network Meta-Analysis for the incidence of pneumothorax.

Four eligible studies (5, 10, 36, 38), comprising 1,267 patients, reported incidence of ARDS, 7 (5, 10, 17, 18, 27, 35, 36) (with 1,383 patients) described incidence of pneumothorax, 4 (5, 10, 29, 36) with a total of 1,368 patients reported incidence of atelectasis, while 4 (5, 10, 36) with 1,255 patients described incidence of hypoxemia. Direct comparison revealed no significant differences among PEEP levels in the various complications (Supplementary Figure 5A). Similarly, Network Meta-Analysis showed that there were no significant differences among the PEEP levels with regards to occurrence of ARDS,atelectasis and hypoxemia (Supplementary Figure 5B), although higher PEEP levels were associated with significantly higher incidence of pneumothorax relative to intermediate and lower PEEP, as well as ZEEP (OR: 2.91e + 12, 95% CI: 40.3, 1.76e + 39; OR: 1.85e + 12, 95% CI: 29.2, 1.18e + 39; and OR: 1.44e + 12, 95% CI: 16.9, 8.70e + 38, respectively) and there was no significant difference among intermediate PEEP, lower PEEP and ZEEP (Figure 9). Node-splitting analysis, based on both direct and indirect comparisons among groups, revealed consistent results (all *p* > 0.05) (Supplementary Figure 6A). Results from ranking analysis showed that high PEEP levels were associated with the highest risk of pneumothorax development, followed by intermediate and lower PEEP, and finally ZEEP (Supplementary Figure 6B).

## Discussion

We employed a Bayesian network meta-analysis to compare ZEEP levels in 28 RCTs (with 2,709 patients) that focused on ventilated patients without ARDS. Our results revealed that intermediate PEEP levels were associated with the highest decrease in the duration of mechanical ventilation, although there were no significant differences among PEEP levels based on direct and indirect comparisons. Meanwhile, higher PEEP levels were associated with significantly higher PFR and increased incidence of pneumothorax. Notably, we found no significant differences among the PEEP groups with regards to LOS of hospital and ICU, hospital, 28-day and ICU mortalities, occurrence of ARDS, atelectasis and hypoxemia. However, our results should be interpreted cautiously, owing to the TSA outcomes and presence of heterogeneity.

One meta-analysis published in 2016 (11) demonstrated that ventilation with higher PEEP levels in ICU patients without ARDS was not associated with neither reduced in-hospital mortality nor shorter ventilation duration, but with lower incidence of ARDS and hypoxemia, as well as higher PaO_2_/FiO_2_. Notably, the study had a moderate to high heterogeneity, while its quality of evidence was low to very low. Consequently, the authors could not address the effects of moderate levels of PEEP (11). When compared to the aforementioned meta-analysis, our study had several strengths. Firstly, we included seven recent studies, which included one large RCT describing use of high PEEP in patients without ARDS. The lower and higher PEEP groups in the former study corresponded to low and intermediate PEEP groups, respectively, in our study (10). This could also explain why our results were not completely consistent with previous meta-analyses. Secondly, we employed a novel classification, and divided the patients into four groups according to the specific PEEP levels. The ZEEP and very high PEEP (>10 cm H_2_O) groups are not routine choices across clinical practice for non-ARDS patients, and these 2 extreme PEEP levels have always been applied in post cardiac surgery patients in our included studies. Moreover, since most of these studies were published 20 years ago, our novel classification allowed us to address the effects of moderate PEEP levels closer to clinical practice. Thirdly, a previous meta-analysis reported PEEP levels that ranged from 0 to10 cm H_2_O for the low group, and 5 to 30 cm H_2_O for the high group, while the heterogeneity was so large that the authors could not make a definite conclusion. Our novel classification solved this problem to a certain extent, and made the conclusion more credible.

Although previous studies have demonstrated the potential benefits and adverse effects of PEEP in ARDS, selecting appropriate PEEP levels seems to be a complex process in patients without ARDS owing to a huge heterogeneity in this population. Although an increase in PEEP levels has been reported in such population in the real-world, evidence of how to choose an optimal concentration was lacking (6, 41). In our study, Bayesian analysis revealed that intermediate PEEP (PEEP = 7–10 cm H_2_O) was associated with shorter duration of mechanical ventilation, whereas network meta-analysis found no significant differences among the studied PEEP levels, which was partially in line with the RELAx trial (10). Interestingly, one study demonstrated that a higher PEEP could reduce the duration of mechanical ventilation (16), was although this corresponded to the intermediate PEEP group in our study. To our knowledge, there were many confounding factors that affected the duration of mechanical ventilation, affirming PEEP's lack of significant impact observed herein.

Our results further showed that PFR was positively correlated with PEEP levels, which was consistent with a previous meta-analysis (11). In ARDS patients, PEEP has been shown to recruit the collapse alveoli, maintain the end expiratory lung volume and improve gas exchange (3). Interestingly, the same principle seems to work in patients without ARDS. On the other hand, inadequate elevated PEEP has been found to cause alveoli overdistension in ARDS patients, thereby causing barotrauma (42, 43). In our opinion, this challenge might be even more pronounced in non-ARDS patients as the collapse alveoli in these patients might be less than those in ARDS patients. This explains why higher PEEP levels were associated with significantly increased incidence of pneumothorax relative to the other PEEP levels in our study. Although the meta-analysis published in 2016 demonstrated that high PEEP was associated with a lower incidence of ARDS and hypoxemia (11), we found no evidence to support this finding.

Although our findings provide evidence of the potential benefits or harmful effects of different PEEP levels, PEEP should not just be applied according to its height, as many physiologic effects of PEEP could be U-shaped (44, 45), Individualized PEEP regimes should be optimized based on a specific patient's physiology rather than focusing simply on the dosage. To date, however, no trial has attempted to evaluate the efficacy of PEEP in patients without ARDS prior to randomization (4), which necessitates future trials.

## Conclusion

In summary, results of our Bayesian network meta-analysis and systematic review revealed that intermediate PEEP levels are associated with the highest decrease in duration of mechanical ventilation in patients without ARDS. However, there were no significant differences among studied PEEP level groups based on both direct and indirect comparisons. Meanwhile, it is evident that higher PEEP levels are associated with significantly higher PFR and increased incidence of pneumothorax. Furthermore, the four studied PEEP levels have no significant impact on LOS of hospital, LOS of ICU, hospital mortality, 28-day mortality, ICU mortality, occurrence of ARDS, as well as atelectasis and hypoxemia.

## Data Availability Statement

The original contributions presented in the study are included in the article/Supplementary Material, further inquiries can be directed to the corresponding author/s.

## Author Contributions

YH and LS: conceptualization, methodology, and supervision. JZ and ZL: data curation and writing-original draft preparation. XD and BL: software and visualization. YZhang, YZheng, HZ, YW, YLai, and WH: writing-review. XL, WH, YX, and YLi: supervision. All authors contributed to the article and approved the submitted version.

## Conflict of Interest

The authors declare that the research was conducted in the absence of any commercial or financial relationships that could be construed as a potential conflict of interest.

## Publisher's Note

All claims expressed in this article are solely those of the authors and do not necessarily represent those of their affiliated organizations, or those of the publisher, the editors and the reviewers. Any product that may be evaluated in this article, or claim that may be made by its manufacturer, is not guaranteed or endorsed by the publisher.
