# Supplementary material for: Optimal Positive End Expiratory Pressure Levels in Ventilated Patients Without Acute Respiratory Distress Syndrome: A Bayesian Network Meta-Analysis and Systematic Review of Randomized Controlled Trials
Source: Front Med (Lausanne). 2021 Sep 1;8:730018. doi: 10.3389/fmed.2021.730018 (PMC8440859; doi:10.3389/fmed.2021.730018)
Supplement: Supplementary file 1 [file Data_Sheet_1.pdf]

SUPPLEMENTARY MATERIALS

Optimal Positive End Expiratory Pressure levels in ventilated patients without Acute Respiratory Distress Syndrome: a Bayesian network meta-analysis and systematic review of randomized controlled trials

Jing Zhou†[1], Zhimin Lin†[1], Xiumei Deng†[1], Baiyun Liu†[1], Yu Zhang[1], Yongxin Zheng[1], Haichong Zheng[1], Yingzhi Wang[1], Yan Lai[1], Weixiang Huang[1], Xiaoqing Liu[1], Weiqun He[1], YuandaXu[1], Yimin Li[1], Yongbo Huang*[1], Ling Sang*[1]

Figure S1 Results of PaO2/FiO2

A.

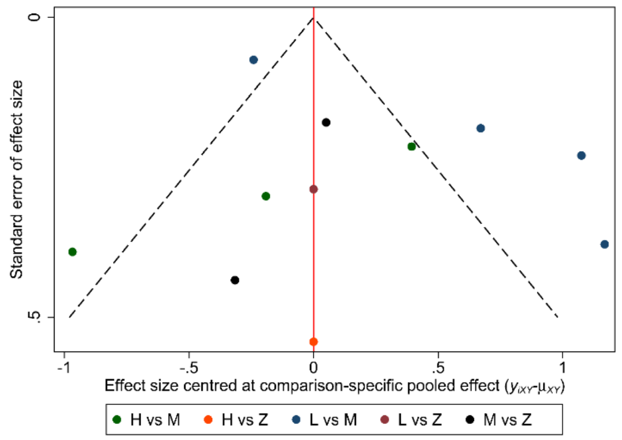

B.

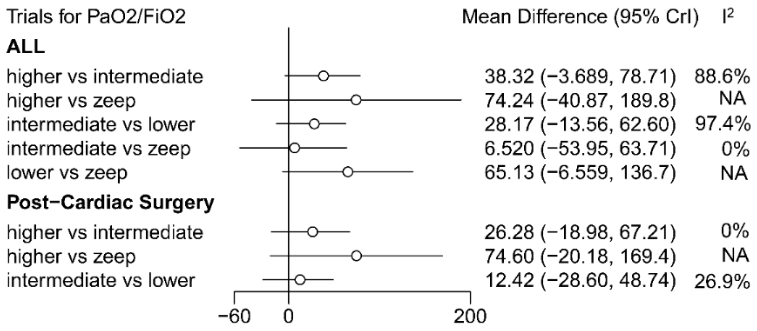

C.

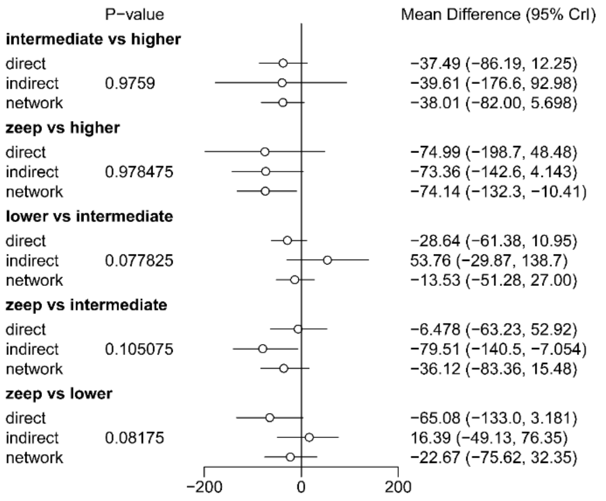

D.

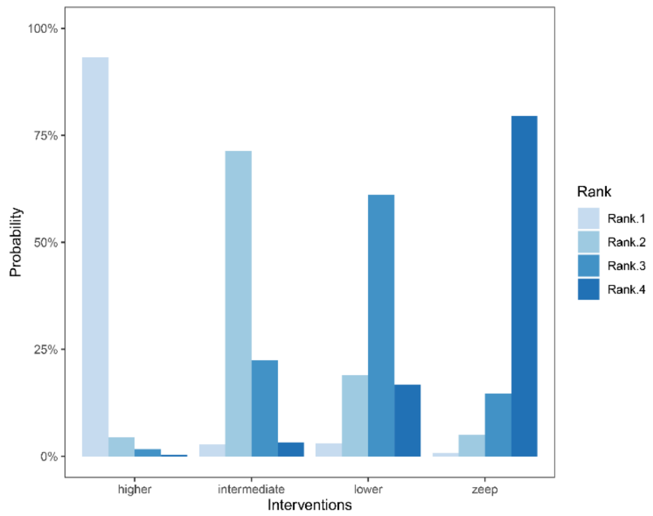

A. Funnel plot of association between estimated effect size for each study **B.** Forest plot of Meta-Analysis in included trials and trials focusing on post-cardiac surgical patients **C.**Node-splitting analysis to assess inconsistency in network meta-analysis (No statistic difference in inconsistency between direct result and indirect result when P-value >0.05). **D.** Cumulative ranking bar graph of each peep level. Ranks represent priority. For each intervention, cumulative ranking bar graph

shows the probabilities when they are at Rank1/2/3/4 respectively. To sum up, the probabilities of every 4 columns in each intervention are 100%.

Figure S2 Results of LOS of hospital

A.

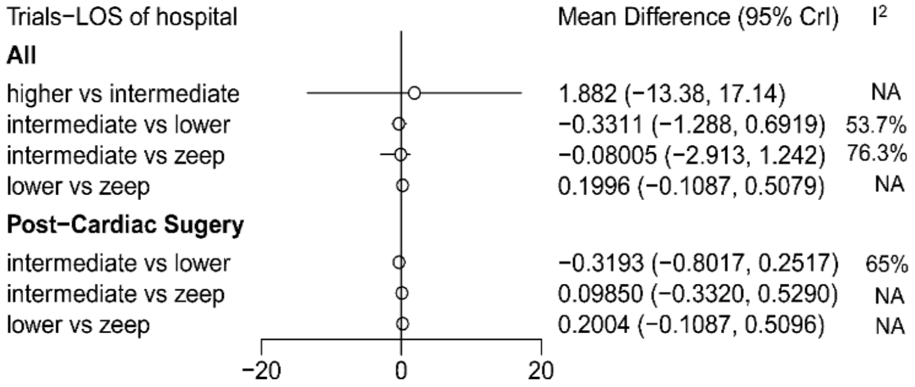

B.

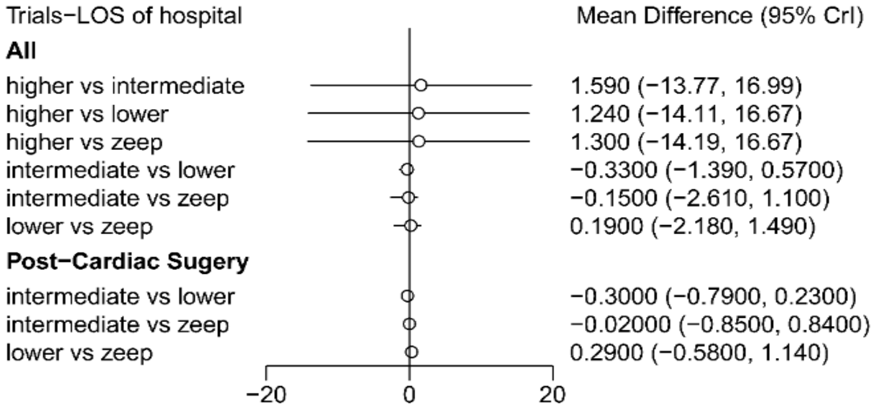

A. Forest plot of Meta-Analysis in included trials and trials focusing on post-cardiac surgical patients **B.** Forest plot of Network Meta-Analysis in included trials and trials focusing on post-cardiac surgical patients. LOS : length of stay

Figure S3 Results of LOS of the ICU

A.

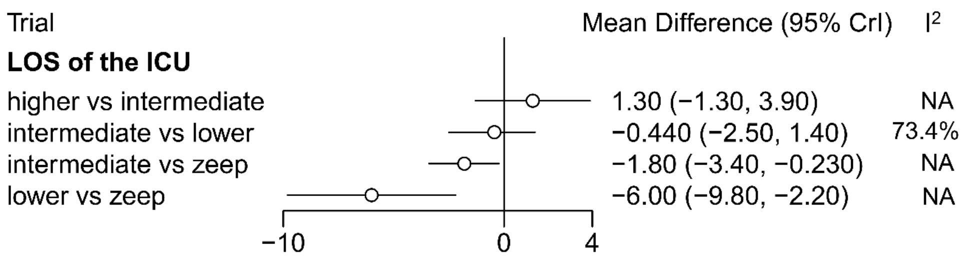

B.

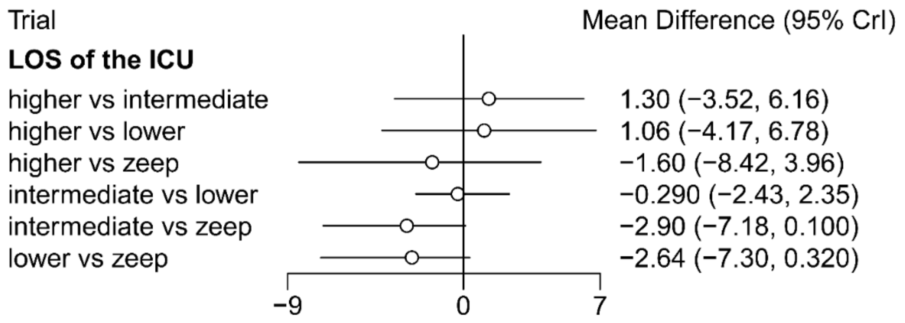

A.Forest plot of Meta-Analysis in included trials **B.** Forest plot of Network Meta-Analysis in included trials.

Figure S4 Results of mortality

A.

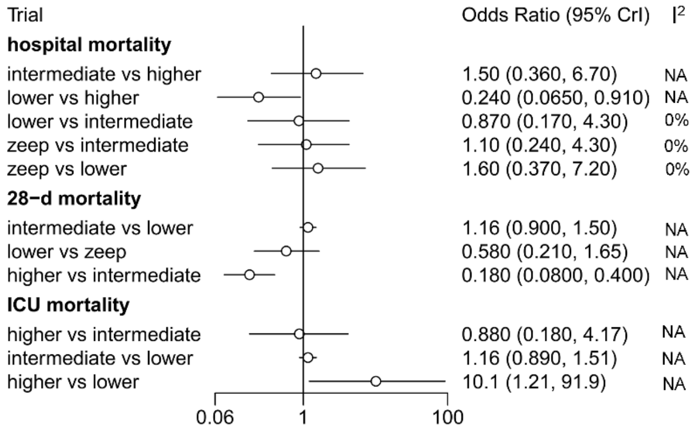

B.

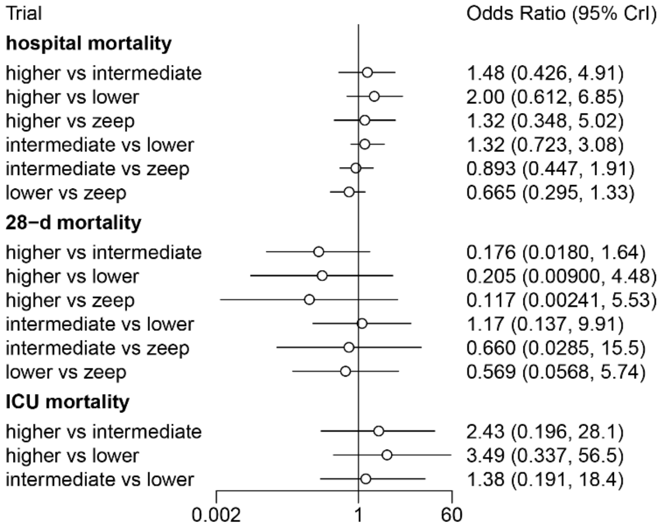

A. Forest plot of Meta-Analysis in included trials **B.** Forest plot of Network Meta-Analysis in included trials

Figure S5 Results of complications

A.

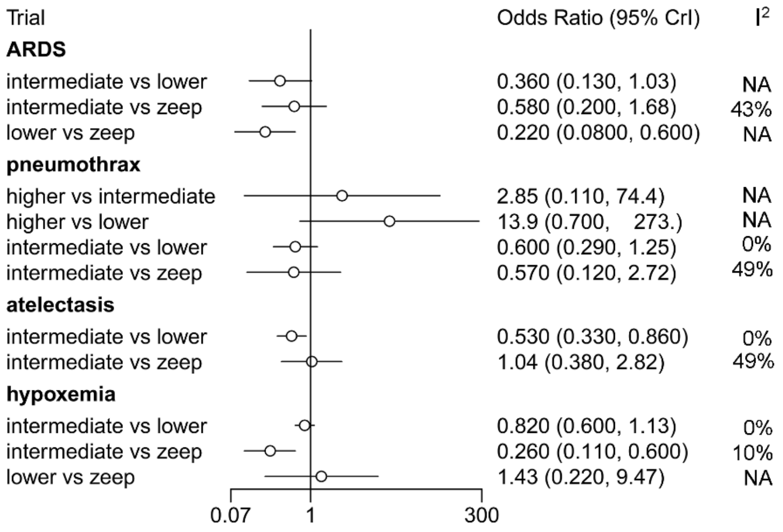

B.

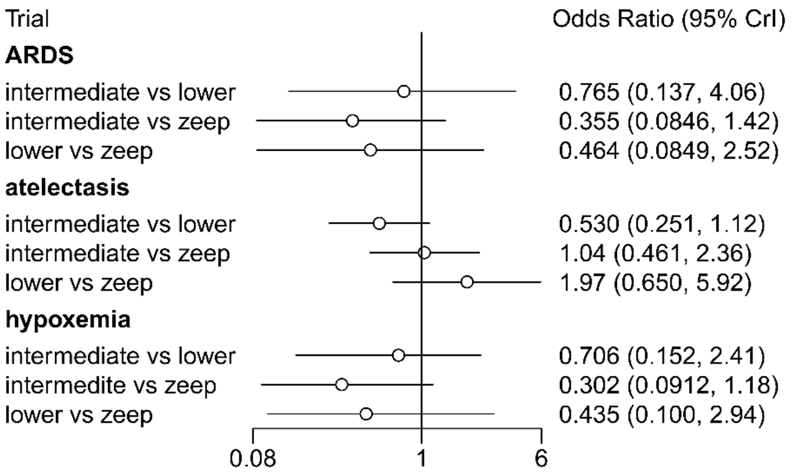

A. Forest plot of Meta-Analysis **B.** Forest plot of Network Meta-Analysis. Complications analyzed in Figure S5 include the incidence of ARDS, pneumothrax, atelectasis and hypoxemia.

Figure S6 Results of complications-the incidence of pneumothorax

A.

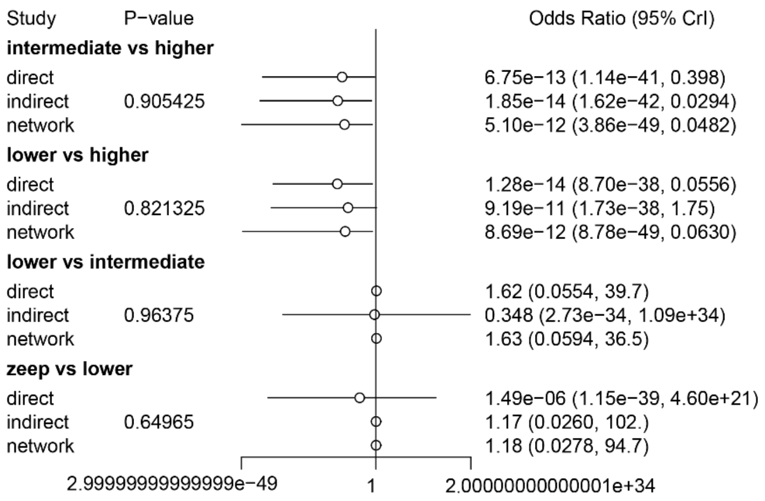

B.

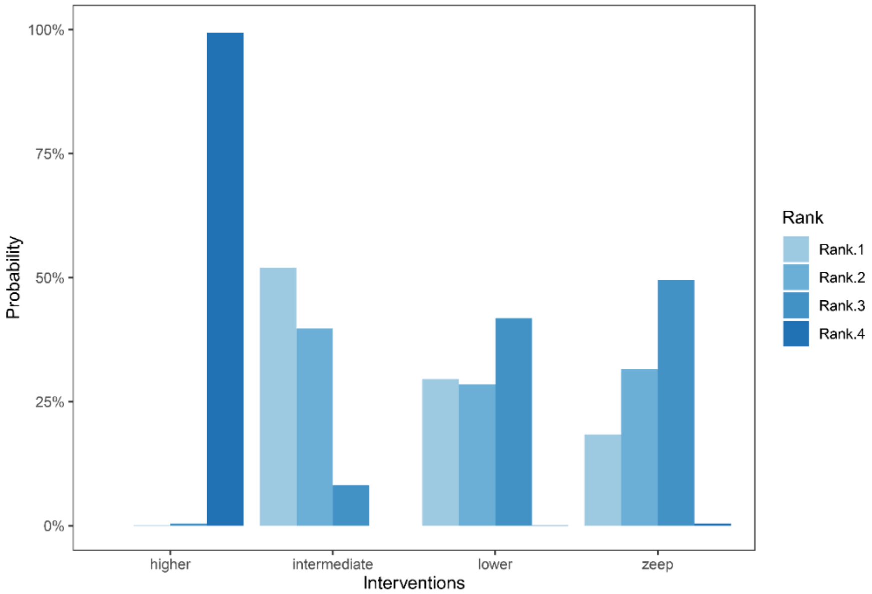

A. Node-splitting analysis to assess inconsistency in network meta-analysis for the incidence of pneumothorax (No statistic difference in inconsistency between direct result and indirect result when P-value >0.05) **B.** Cumulative ranking bar graph of each peep level. Ranks represent priority. For each intervention, cumulative ranking bar graph shows the probabilities when they are at Rank1/2/3/4 respectively. To sum up, the probabilities of every 4 columns in each intervention are 100%.
